# Improved Beam Angle Arrangement in Intensity Modulated Proton Therapy Treatment Planning for Localized Prostate Cancer

**DOI:** 10.3390/cancers7020574

**Published:** 2015-03-30

**Authors:** Wenhua Cao, Gino J. Lim, Yupeng Li, X. Ronald Zhu, Xiaodong Zhang

**Affiliations:** 1 Department of Industrial Engineering, University of Houston, Houston, TX 77204, USA; E-Mails: wcao@central.uh.edu (W.C.); ginolim@central.uh.edu (G.J.L.); 2 Applied Research, Varian Medical Systems, Palo Alto, CA 94304, USA; E-Mail: Yupeng.Li@varian.com; 3 Department of Radiation Physics, The University of Texas M. D. Anderson Cancer Center, Houston, TX 77030, USA; E-Mail: xrzhu@mdanderson.org

**Keywords:** IMPT, treatment planning, beam angle optimization, prostate cancer

## Abstract

*Purpose*: This study investigates potential gains of an improved beam angle arrangement compared to a conventional fixed gantry setup in intensity modulated proton therapy (IMPT) treatment for localized prostate cancer patients based on a proof of principle study. *Materials and Methods*: Three patients with localized prostate cancer retrospectively selected from our institution were studied. For each patient, IMPT plans were designed using two, three and four beam angles, respectively, obtained from a beam angle optimization algorithm. Those plans were then compared with ones using two lateral parallel-opposed beams according to the conventional planning protocol for localized prostate cancer adopted at our institution. *Results*: IMPT plans with two optimized angles achieved significant improvements in rectum sparing and moderate improvements in bladder sparing against those with two lateral angles. Plans with three optimized angles further improved rectum sparing significantly over those two-angle plans, whereas four-angle plans found no advantage over three-angle plans. A possible three-beam class solution for localized prostate patients was suggested and demonstrated with preserved dosimetric benefits because individually optimized three-angle solutions were found sharing a very similar pattern. *Conclusions*: This study has demonstrated the potential of using an improved beam angle arrangement to better exploit the theoretical dosimetric benefits of proton therapy and provided insights of selecting quality beam angles for localized prostate cancer treatment.

## 1. Introduction

The adoption of proton therapy for localized prostate cancer treatment has increased rapidly in recent years as more proton facilities have become available for patient care. However, recent cohort studies [1,2,3] on localized prostate cancer patients who had received proton therapy revealed that proton therapy is not superior to photon-based intensity modulated radiation therapy (IMRT) in terms of reducing radiation-induced toxicity. One study [3] on nationally sampled data indicated that there is no statistically difference between proton therapy and IMRT regarding genitourinary, gastrointestinal, and other treatment-related toxicity. Two other studies [1,2] on single-institution data found that proton therapy is associated with more gastrointestinal morbidity than IMRT. Note that the lager volume of rectum receiving excessive dose, especially in high dose levels, is strongly correlated with post-treatment genitourinary or gastrointestinal toxicity [4,5,6,7]. It also should be noted that the data used in those studies are based on the older technology of proton therapy, *i.e.*, passive scattering proton therapy (PSPT) [8,9]. The resultant findings not only suggest the necessity for more thorough comparative studies between PSPT and its alternatives on patient quality of life after treatment, but also demonstrate a dire need for further improvement of proton therapy including better rectum sparing particularly. In fact, selected facilities has begun using the more advanced intensity modulated proton therapy (IMPT) with expected higher benefits in target volume coverage and healthy tissue sparing over PSPT in clinical treatment for localized prostate cancer [10,11,12,13].

However, most localized prostate cancer patients are treated by two parallel-opposed lateral beams according to the clinical PSPT or IMPT treatment planning protocol commonly used in proton facilities [8,10]. This standard beam angle arrangement was originally introduced to diminish the impact of range uncertainties of protons [14,15] during the initial development of clinical proton therapy. Yet, this approach not only cancels the benefits of using distal fall off of proton deposited dose (or Bragg peak [16]) to spare critical structures (rectum or bladder) but also can cause higher scatter and wider dose penumbra [8]. Along with recent researches on applying robust optimization to diminish treatment uncertainty in the planning process [17,18,19,20] or adopting active proton range control in the delivering process [21,22], unconventional beam angles (other than the two parallel-opposed lateral ones) could be safely used. Initial studies [8,22,23] showed that substantially improved rectum sparing was achieved by oblique angles compared to lateral angles.

It is a good time now to revisit the question: “what is the best beam angle arrangement for proton therapy localized prostate cancer treatment?” The current standard of beam angles is insufficient to support proton therapy as a more effective radiotherapy compared to its alternatives. In this paper, we investigate the potential of employing improved beam angle arrangements to exploit higher advantages of proton therapy based on a proof of principle study. The preferred number and setup of beam angles are then recommended as a pointer for future development of proton therapy for treating localized prostate cancer.

## 2. Materials and Methods

Three random patients with localized prostate cancer were retrospectively selected from our institution in this study. Arrangements of two, three and four beam angles for each of the three patients were determined by a beam angle optimization algorithm [23]. Those optimized beam angle arrangements were compared to the conventional two lateral-opposed angles (90°, 270°) by evaluating the resultant IMPT plans based on multi field optimization [10]. The prescribed dose to the planning target volume (PTV) was set at 78 Gy (relative biological equivalence [RBE]) for 39 fractions for all patients. The IMPT plans were optimized by the Eclipse treatment planning system (Varian Medical Systems, Inc., Palo Alto, CA, USA), and normalized with 97.5% prescribed dose for the PTV.

The optimized beam angle arrangements obtained from the optimization algorithm [23] for two, three, and four angles were (90°, 310°), (10°, 140°, 270°), and (10°, 140°, 270°, 310°) for patient 1; (40°, 290°), (0°, 150°, 270°), and (20°, 140°, 200°, 310°) for patient 2; (40°, 230°), (40°, 150°, 270°), and (40°, 90°, 210°, 350°) for patient 3. Although all optimized beam angle arrangements are patient specific, the three-angle arrangements are very characteristic. The right left lateral beam 270° was shown for all three cases. The left posterior oblique beam 150° was shown for two patients and 140° for one patient. The anterior right oblique beams, *i.e.*, 0°, 10°, 40° were shown for each of the three patients respectively. This result indicated that it might be possible to design a class solution of beam angles for different prostate patients. Based on our cross validations among the three patients, we found that angles 270°, 10° and 140° could lead to good IMPT plans consistently. Therefore, a class three-angle arrangement (10°, 140°, 270°) was also used and compared to the conventional two-angle arrangement. In addition, IMPT plans with the single field uniformly distribution (SFUD) [10] and well-designed photon volumetric modulated arch therapy (VMAT) plans [24] for the three patients were also used in dosimetric comparisons.

## 3. Results

The average rectum and bladder volumes receiving doses of 30, 40, 50, 60, and 70 Gy (RBE), and mean doses for all three patients are listed in Table 1. Overall, the optimized two, three and four angles all achieved better sparing of the rectum and the bladder than the two parallel-opposed lateral beams. While the optimized two-angle plans improved rectum and bladder sparing by a mean dose reduction of 16.9% for the rectum and 0.6% for the bladder, the optimized three-angle plans further improved rectum sparing by a mean dose reduction of 25.6% and reached the same bladder sparing as the optimized two-angle plans. However, the optimized four angles failed to achieve further improvement on both rectum and bladder sparing, which were instead less good than the three angles.

**Figure 1:**
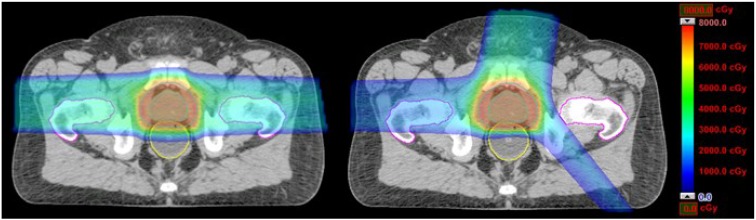
Dose distribution plots for the conventional two-angle IMPT plans (90°, 270°) and the class three-angle IMPT plans (10°, 140°, 270°) for one prostate cancer patient.

Table 1 shows that at most 5% variation in dose volume data between the optimized and class three-beam-angle plans were observed. This indicates that the advantages of the optimized beam angles could be closely represented by a class three-angle arrangement. The volumes of the rectum receiving doses of 30, 40, 50, 60, and 70 Gy (RBE) and the mean rectum dose in plans with the conventional two beam angles were reduced by 29.6%, 29.7%, 25.7%, 23.1%, 15.9%, and 21.5%, respectively, by plans with the three class beam angles. On the other hand, the dose volumes of the bladder were reduced at −0.4%, 0.5%, 1.9%, 3.8%, 5.3%, 6.7%, and 0.0%, respectively. For illustration, dose distributions of the conventional two-angle plan and the class three-angle plan in an identical transversal plane for patient 1 are shown in Figure 1.

**Table 1:** Dose volume data for IMPT plans with different beam angle arrangements in average by three prostate cancer patients.

ROI	Statistic *	Two Angles (Conventional)	Two Angles (Optimized)	Three Angles (Optimized)	Four Angles (Optimized)	Three Angles ** (Class)	
Rectum	V_30Gy_ (%)	22.6	16.8	15.7	16.0	15.9	(29.6%)
	V_40Gy_ (%)	18.2	13.9	12.6	13.2	12.8	(29.7%)
	V_50Gy_ (%)	14.4	11.3	10.2	10.7	10.7	(25.7%)
	V_60Gy_ (%)	10.8	8.9	8.0	8.4	8.3	(23.1%)
	V_70Gy_ (%)	6.9	6.2	5.7	5.7	5.8	(15.9%)
	D_mean_ (Gy)	17.2	14.3	12.8	13.7	13.5	(21.5%)
Bladder	V_30Gy_ (%)	22.7	22.5	22.6	23.7	22.9	(−0.4%)
	V_40Gy_ (%)	19.3	18.7	18.7	19.4	19.2	(0.5%)
	V_50Gy_ (%)	16.2	15.5	15.5	15.9	15.9	(1.9%)
	V_60Gy_ (%)	13.1	12.3	12.4	12.8	12.6	(3.8%)
	V_70Gy_ (%)	9.4	8.8	8.8	9.2	8.9	(5.3%)
	D_mean_ (Gy)	17.2	17.1	17.1	17.5	17.2	(0.0%)

* All dose volume indices are based on dose in Gy (relative biological equivalence [RBE]); ** The reductions of dose volume data for class three-angle plans comparing to conventional two-angle plans are provided in parentheses.

**Figure 2:**
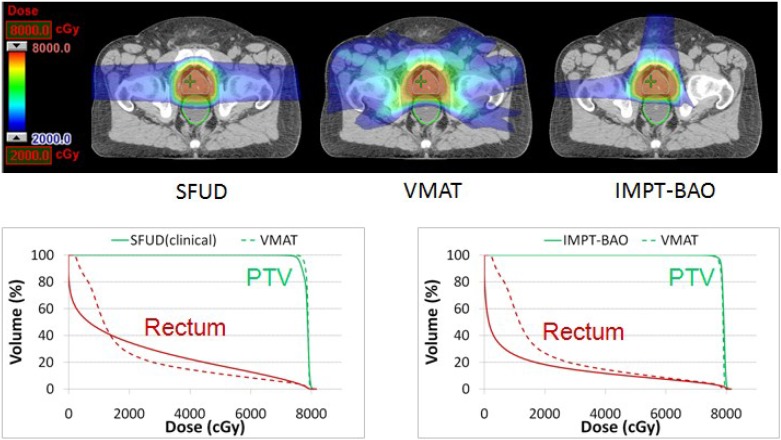
Comparison of SFUD, VMAT and IMPT treatment plans for one prostate cancer patient. Here SFUD represents a two-beam IMPT single field uniform dose distribution plan. This plan is used to treat patients at our institution. VMAT represents a photon volumetric modulated arch therapy plan. IMPT-BAO represents a class three-angle IMPT plan, where BAO interprets beam angle optimization.

The class three-angle plans were also compared with clinical SFUD plans and VMAT plans. As shown in Figure 2, the rectum sparing of the IMPT SFUD plan with two lateral beams is inferior to the VMAT plan [24]. However, if we compare the three-beam IMPT plan suggested in this work with the VMAT plan, we can see that IMPT plan is superior to VMAT plan in terms of rectum sparing with similar PTV coverage. Note that we found no marked difference in the bladder sparing among three plans. This example demonstrated that it is important to explore the appropriate number of beams and the selection of beam angles to fully take advantages of IMPT. The patient studies also demonstrated that the directions toward the OARs might have to be used to design the better proton plan.

## 4. Discussion

In clinical proton therapy treatments, the same set of two parallel-opposed lateral beam angles are used for localized prostate cancer patients. This is caused mainly by two considerations: (1) geometrical relationships between the target and the organs at risk (OARs) are relatively invariable from one patient to another; (2) those two lateral angles other than oblique angles can avoid aiming at the rectum or bladder directly so that the effect of proton range uncertainties can be minimized. However, the best property of proton beam is its distal fall-off which enables proton therapy to achieve precise dose delivery with maximum sparing of OARs and normal tissues. This theoretical benefit of proton therapy is greatly sacrificed in prostate treatment by the current beam angle arrangement because the OARs including rectum and bladder are entirely spared by lateral edges rather than distal edges of the two lateral beams. Recently, a great deal of studies on both robust treatment plan optimization [17,18,19,20,25,26] and imaging-based proton beam range verification [21,22,27,28] have been conducted to compensate the effect of uncertainties in proton therapy. Therefore, it is now important to improve the beam angle arrangement to better exploit the therapeutic advantages of proton therapy. The investigation in this paper demonstrated promising potentials of using improved beam angle arrangements in OAR sparing. For example, reductions of as much as 26%, 17% and 26% in rectum V60, V70, and mean dose were achieved by optimized three-angle plans comparing to the conventional two-angle plans (Table 1).

We observed that the three-angle arrangements achieved the best IMPT plan quality among angle arrangements in different numbers for all three prostate cancer patients. More than three angles failed in leading to an improved plan quality. Therefore, the best judgment call we can raise is that three beam angles can be the minimum number of treatment beam angles to achieve quality plans for other prostate cancer patients. We chose three the most separate angles from those individually optimized three-angle arrangements, *i.e.*, (10°, 140°, 270°), to be the class solution of three beam angles for common clinical prostate cancer treatment. As illustrated in Table 1, the class three-angle plans performed very similarly to the optimized three-angle plans in all dosimetric measurements.

In principle, the improved beam angle arrangements are readily applicable to clinical treatment. If the IMPT treatment does not include proactive proton range control methods such as online field-specific range verification and adjustment [22], plan robustness with respect to treatment uncertainties [29] including ones presumably introduced by certain “non-robust” angles can also to be managed during treatment planning process. One useful approach is the robust analysis of an optimized IMPT plan. For example, we compared worst-case dose distributions [19] of the conventional two-angle and class three-angle plans for the three patients and dose volume data are listed in Table 2.

**Table 2:** Dose volume data for IMPT plans with conventional two beams (90°, 270°) and class three beams (10°, 140°, 270°) for three prostate cancer patients in the worst-case scenario.

ROI	Statistic *	Case 1		Case 2		Case 3	
		Two-Beam	Three-Beam	Two-Beam	Three-Beam	Two-Beam	Three-Beam
Rectum	V_30Gy_ (%)	31.1	26.8	29	25.5	35.1	30.1
	V_40Gy_ (%)	25.7	20.4	25	21.1	29.6	25.8
	V_50Gy_ (%)	20.9	16.3	21	17.2	24.6	21.7
	V_60Gy_ (%)	16.3	12	17	13.3	19.5	16.2
	V_70Gy_ (%)	11.5	8.8	13	10.8	14.5	12.3
	D_mean_ (Gy)	25.2	20.1	24.8	20.3	27.2	24.5
Bladder	V_30Gy_ (%)	17.3	17.5	27.1	27.8	30.3	30.7
	V_40Gy_ (%)	14.6	14.9	23.3	23.3	27	26.8
	V_50Gy_ (%)	12.3	12.4	19.9	19.9	23.8	23.2
	V_60Gy_ (%)	10.1	10.5	16.4	16.8	20.4	20.5
	V_70Gy_ (%)	7.7	7.7	12.4	12.5	16.3	16.2
	D_mean_ (Gy)	13.6	13.8	21.2	21.3	25.8	25.4

* All dose volume indices are based on dose in Gy (relative biological equivalence [RBE]).

**Figure 3:**
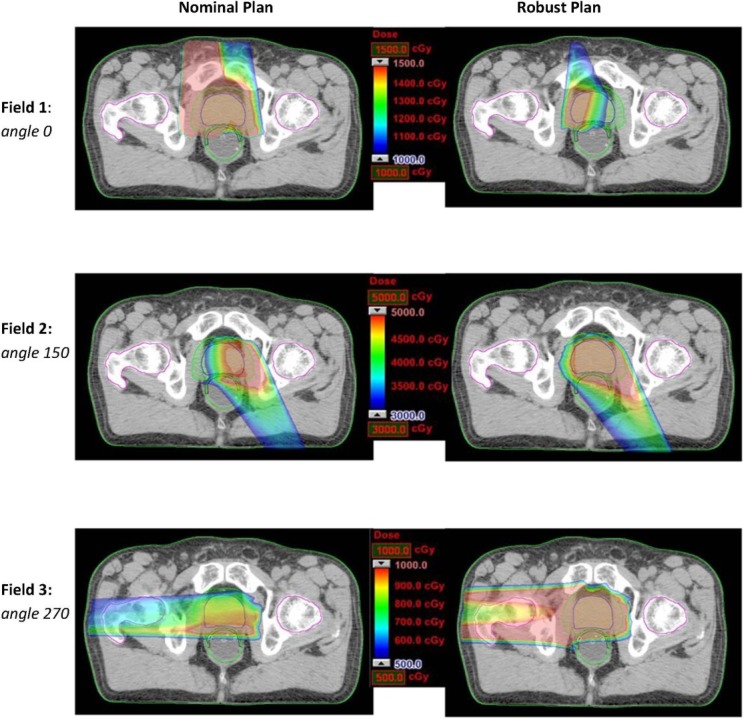
Dose distributions (per field) in the transverse plan for comparing the nominally optimized and robustly optimized three-beam IMPT plan for one prostate cancer patient.

**Figure 4:**
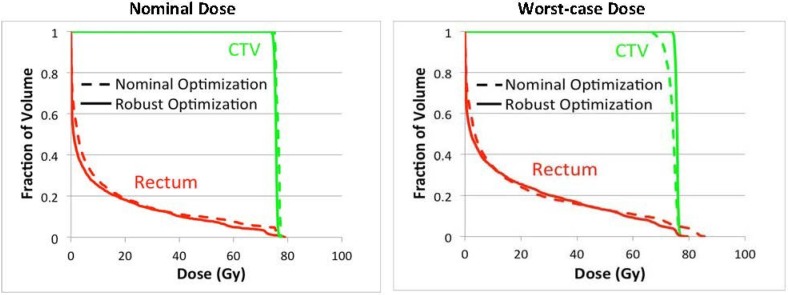
Dose (RBE) volume histograms for CTV and rectum from nominal and worst-case dose distributions based on nominally and robustly optimized plans with three beams (0°, 150°, 270°) for one prostate cancer patient.

Under the worst-case scenario, the significant reduction of dose on rectum by three-beam plans comparing to two-beam plans was consistently preserved. This indicates the three-angle plans are still preferable upon the realization of certain uncertainties. In addition, one can also apply robust optimization for mitigating uncertainty effects. Due to the high flexibility of IMPT, intensity modulation of proton beams can enable incident fields to produce individually inhomogeneous dose distributions while their combination forming a homogeneous and robust dose distribution. Figure 3 shows an example of a robustly optimized three-beam plan (patient 2) using a robust optimization algorithm [25] in comparison with a nominally optimized plan. The robust plan tends to limit dose coverage by the beam incident from the zero-degree gantry angle, while providing more uniform dose coverage by angles 150° and 270°. In contrast, the nominal plan includes more uniform dose coverage by angle 0°. Ultimately, the sensitivity to uncertainties can be mitigated from the nominally optimized to the robustly optimized plan for the same set of treatment beam angles. Figure 4 shows the dose volume histograms for the clinical target volume (CTV) and the rectum from nominal and worst-case dose distributions based on nominally and robustly optimized plans for the same patient (shown in Figure 3).

The most important advantage of using the improved beam angle arrangements is the reduction of rectal doses in localized prostate cancer treatment. Recent comparative studies [1,2,3] on patient data available to date for the early after-treatment period claimed that proton therapy is no better than the currently most-adopted IMRT regarding after-treatment rectal complications. It is believed that there is a dominant connection between rectal doses and rectal toxicity [5,6,7] and bleeding [4,30]; and the higher risk develops as the larger volumes of rectum receiving higher doses, e.g., doses greater than 60 Gy (RBE) [5]. In this study, the class three-beam arrangement was able to reduce the percentage of volume of rectum receiving doses of 60 and 70 Gy (RBE) by 23.1% and 15.9% averagely for the three prostate patients. Therefore, improving beam angle arrangement is a direct approach to sustain proton therapy as an attractive and viable treatment option for prostate cancer. Since there are more the investment-intensive proton facilities either in operation or under development, it is more urgent to improve treatment quality of proton therapy. In future study, we will thoroughly validate the feasibility of incorporating improved beam angle arrangements into the IMPT prostate treatment workflow at our center so that a clinical routine can be possibly established.

## 5. Conclusions

Theoretical advantages of proton therapy have not been fully translated to clinical benefits in localized prostate cancer treatment due to the limited use of proton distal fall-off to spare critical structures in the current beam angle arrangement strategy. This study investigated the potential gains of using improved beam angle arrangements compared to the conventional two bilateral angles for IMPT treatment and provided insights of selecting quality beam angles for prostate cancer patients. A possible three-angle class solution was suggested to significantly improve rectum sparing comparing to the conventional two-angle approach. Promising findings of using unconventional beam angles will ultimately help guide future improvement in IMPT plan quality.
